# Repeat-dose toxicity study of human umbilical cord mesenchymal stem cells in cynomolgus monkeys by intravenous and subcutaneous injection

**DOI:** 10.3389/fcell.2023.1273723

**Published:** 2023-10-25

**Authors:** Wei Pan, Liqiang Gu, Hongzhong Yang, Cong Xu, Zhengbiao Yang, Qijiong Lu, Yuhua Shi, Lili Zhang, Jinjin Shao, Yunxiang Chen, Xin Pan, Feifei Wu, Ruolang Pan, Jinfeng Liang, Lijiang Zhang

**Affiliations:** ^1^ Key Laboratory of Drug Safety Evaluation and Research of Zhejiang Province, Center of Safety Evaluation and Research, Hangzhou Medical College, Hangzhou, China; ^2^ Engineering Research Center of Novel Vaccine of Zhejiang Province, Hangzhou Medical College, Hangzhou, China; ^3^ Zhejiang Key Laboratory of Cell‐Based Drug and Applied Technology Development, S-Evans Biosciences Co, Ltd., Hangzhou, China; ^4^ Zhejiang Center for Drugs and Cosmetics Evaluation, Zhejiang Province Food and Drug Administration, Hangzhou, China

**Keywords:** umbilical cord mesenchymal stem cells, repeat-dose toxicity, cynomolgus monkeys, immunosuppression, intravenous and subcutaneous injection

## Abstract

Human umbilical cord mesenchymal stem cells (hUC-MSCs) are proposed for the treatment of acute lung injury and atopic dermatitis. To advance hUC-MSC entry into clinical trials, the effects of hUC-MSCs on the general toxicity, immune perturbation and toxicokinetic study of hUC-MSCs in cynomolgus monkeys were assessed. hUC-MSCs were administered to cynomolgus monkeys by intravenous infusion of 3.0 × 10^6^ or 3.0 × 10^7^cells/kg or by subcutaneous injection of 3.0 × 10^7^cells/kg twice a week for 3 weeks followed by withdrawal and observation for 6 weeks. Toxicity was assessed by clinical observation, clinical pathology, ophthalmology, immunotoxicology and histopathology. Moreover, toxicokinetic study was performed using a validated qPCR method after the first and last dose. After 3rd or 4th dosing, one or three the monkeys in the intravenous high-dose group exhibited transient coma, which was eliminated by slow-speed infusion after 5th or 6th dosing. In all dose groups, hUC-MSCs significantly increased NEUT levels and decreased LYMPH and CD3^+^ levels, which are related to the immunosuppressive effect of hUC-MSCs. Subcutaneous nodules and granulomatous foci were found at the site of administration in all monkeys in the subcutaneous injection group. Other than above abnormalities, no obvious systemic toxicity was observed in any group. The hUC-MSCs was detectable in blood only within 1 h after intravenous and subcutaneous administration. The present study declared the preliminary safety of hUC-MSCs, but close monitoring of hUC-MSCs for adverse effects, such as coma induced by intravenous infusion, is warranted in future clinical trials.

## 1 Introduction

Mesenchymal stem cells (MSCs) are adult stem cells with self-renewal ability, high proliferation capacity and multidirectional differentiation potential that have attracted much attention for their therapeutic potential (Naji, Eitoku, Favier et al., 2019; Hoang, Pham, Bach et al., 2022). The preclinical and clinical studies suggest that MSCs have therapeutic potential in heart failure, spinal cord injury, and ischemic stroke by repairing and regenerating damaged tissues and organs (Bartolucci, Verdugo, González et al., 2017; Cofano, Boido, Monticelli et al., 2019; Han et al., 2019; Chung, Chang, Bang et al., 2021). Apart from their role in regenerative medicine, MSCs exert immunosuppressive functions by inhibiting T-cell proliferation, generating regulatory T (Treg) cells, and reducing inflammatory factor release, such as tumor necrosis factor-α (TNF-α) and interferon-γ (IFN-γ) (Shi et al., 2011; Yi and Song, 2012; Chao et al., 2014; Wang et al., 2018; Song et al., 2020). MSCs have been widely used to treat immune mediator-related diseases including GVHD, inflammatory bowel disease, rheumatoid arthritis and sepsis. Human umbilical cord mesenchymal stem cells (hUC-MSCs) are isolated from the umbilical cord and have the advantages of low immunogenicity, rich sources, convenient collection, and lack of ethical issues compared to other stem cells (Deuse, Stubbendorff, Tang-Quan et al., 2011; Zhang, Tao, Liu et al., 2017). Therefore, hUC-MSCs have attracted considerable interest in various biological fields, especially in immune disease therapy (Abbaspanah et al., 2021). Our previous study showed that hUC-MSC transplantation protected the lipopolysaccharide-induced acute lung injury mouse model and TNCB or OVA-induced atopic dermatitis (AD) rat model (Ren, Zhang, Wang et al., 2018; Cui, Zhang, Pan et al., 2022).

The route of transplantation of MSCs has an important impact on the therapeutic efficacy. The intravenous route is the most commonly used route in animal experiments and clinical trials. However, the majority of stem cells delivered by the intravenous route become trapped in the lung, spleen and liver and have difficulty reaching target organs. Increasing evidence has shown that the locoregional delivery of stem cells by intramuscular, intra-arterial, intradermal route, etc., can improve the therapeutic efficacy. A clinical trial proved that the subcutaneous injection of hUC-MSCs resulted in dose-dependent improvements of disease index score in atopic dermatitis (AD) patients (Matthay, Goolaerts, Howard et al., 2010; Kim, Lee, Roh et al., 2017; Kim, Lee, Kim et al., 2018; Chen et al., 2019). Although some studies on the safety evaluation of hUC-MSCs have been reported, we still require more scientific data to evaluate the risk of hUC-MSCs therapy, including its general toxicity, toxicokinetic and immune perturbation. Additionally, safety studies of the subcutaneous delivery of hUC-MSC therapies have not been reported before. Here, we evaluated the safety and toxicity of hUC-MSCs in cynomolgus monkeys via intravenous and subcutaneous routes to obtain detailed safety data. Our preliminary test results showed the low toxicity and immunosuppressive effect of hUC-MSCs.

## 2 Materials and methods

### 2.1 hUC-MSCs preparation

The hUC-MSCs and cell diluent were manufactured and provided by S-Evans Biosciences Co., Ltd. (Hangzhou, China). In brief, an umbilical cord tissue was obtained from a healthy pregnant woman which was approved by Ethics Committee of the 2nd Affiliated Hospital, School of Medicine, Zhejiang University (NO. 2021-1135). The tissue was cut into approximately 1 mm^3^ pieces for primary adherent culturing. When the cells reached 80%–90% confluence, they were digested with 0.25% trypsin-EDTA for harvesting and passaging. hUC-MSCs at passage 5 were used for the present research. As quality control, cells were characterized by morphology, mesenchymal lineage differentiation and surface marker expression. The capacity of osteogenic, adipogenic, and chondrogenic mesenchymal lineage differentiation was detected using Alizarin Red, Oil Red O, and Alcian Blue staining (OriCell, Guangzhou, China), respectively. Surface marker expression was also characterized by flow cytometry with CD73, CD90, CD105, CD34, CD14, CD19, CD45, and HLA-DR antibodies (BD Bioscience, San Jose, CA, United States) as described by previous study (Li et al., 2021; Liu, Song, Huang et al., 2021). Cells were 95% viable by trypan blue exclusion. The cells were freshly prepared on the day of use and resuspended in saline supplied with 2% Human albumin (Octapharma, Germany) and placed on ice until transplantation within 6 h after preparation.

### 2.2 Animals

A total of 40 cynomolgus macaques aged from 3.8 to 4.1 years and weighing from 2.53 to 3.67 kg, half male and half female, were purchased from Hainan JinGang Biotechnology Co., Ltd, China (Laboratory Animal Production License No: SCXK (Qiong) 2015-0001, Laboratory animal quality certificate No.460012000000413 and No.460012000000396. Animals were housed in stainless steel cages in a ventilated animal room at 20∼24°C with HEPA-filtered air at a rate of 8∼10 air changes per hour and a humidity of 57%∼69% under a 12:12-h light-to-dark cycle. Animals were fed *ad libitum*. The number of animals used in each group meets the requirements of the NMPA’s Technical Guidelines for Repeated-dosing Toxicity Studies of Drugs, which call for 10 non-rodents per group, half male and half female (Center for Drug Evaluation and NMPA, 2014). This study was approved by the Institutional Animal Care and Use Committee (IACUC) of Center of Safety Evaluation and Research, Hangzhou Medical College under protocol number GLP-2021-017 based on the 3R principle (Reduction, Replacement and Refinement). All animal experimental protocols and welfare protocols complied with the related ethics regulations. The animal facility was fully AAALAC accredited (AAALAC, #001489). This study was conducted in accordance with Good Laboratory Practices (GLP). Considering that cynomolgus monkeys are non-human primates, and has the closest genetic relationship to humans, the present nonclinical study was conducted in cynomolgus monkeys with repeated administration.

### 2.3 Study design

**FIGURE 1:**
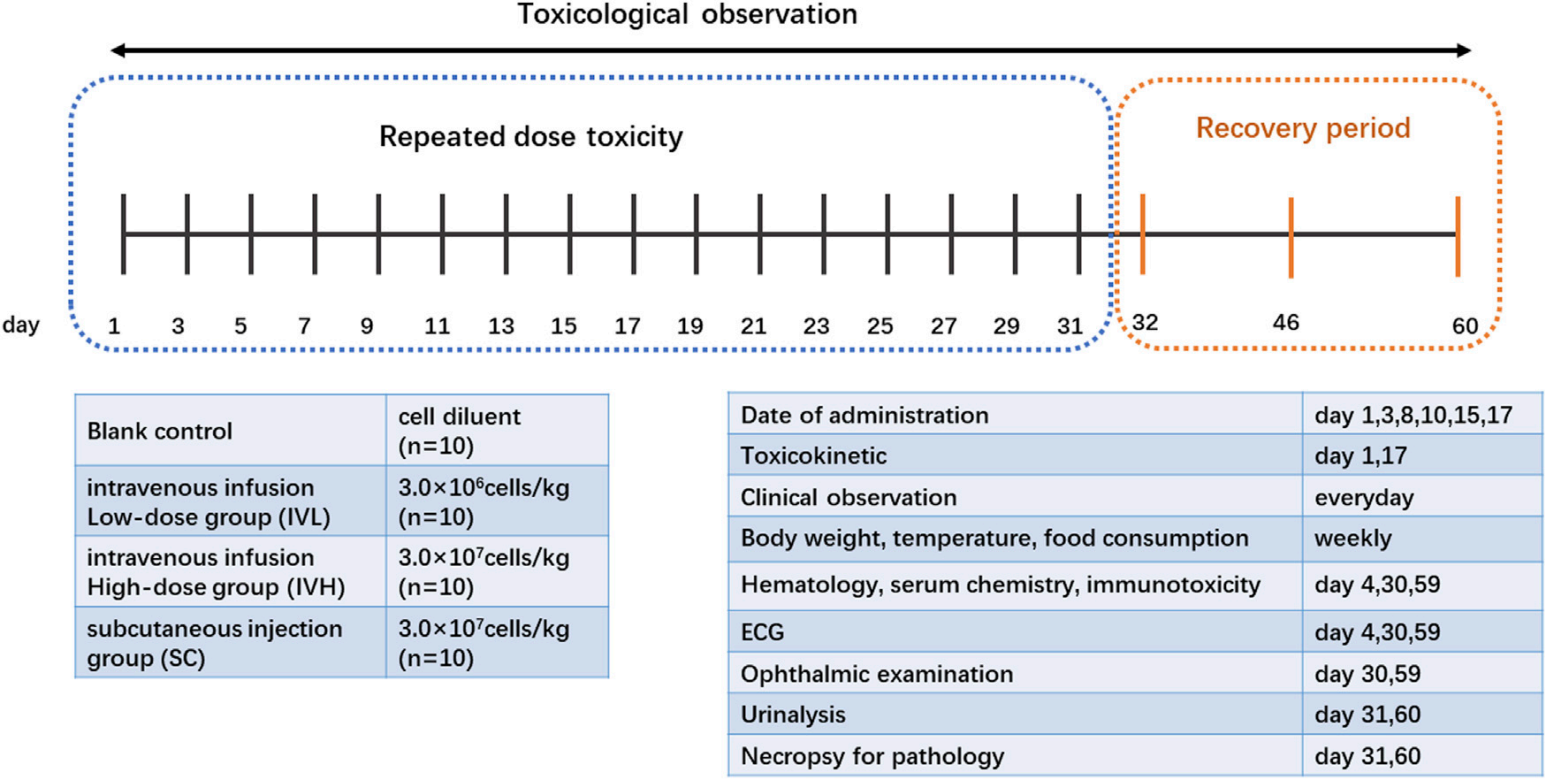
Experimental design and scheme of the repeated-dose toxicity study for hUC-MSCs. Cynomolgus monkeys were divided into 4 dosing groups: 0, 3.0 × 10^6^, and 3.0 × 10^7^cells/kg intravenously or 3.0 × 10^7^cells/kg subcutaneously, *n* = 10 per group, and hUC-MSCs were administered twice a week for 3 weeks.

Forty cynomolgus macaques were randomized into four groups (*n* = 10) as follows: control group, intravenous infusion low-dose group (IVL), intravenous infusion high-dose group (IVH), and subcutaneous injection group (SC). The control group received 2.5 mL/kg cell diluent, the IVL and IVH groups received 3.0 × 10^6^ or 3.0 × 10^7^ cells/kg hUC-MSCs intravenously, and the SC group received 3.0 × 10^7^ cells/kg hUC-MSCs subcutaneously into the dorsal skin. The dose levels of hUC-MSCs were selected based on the proposed adult clinical dose (2.0 × 10^6^ cells/kg for intravenous use, 1.0 × 10^6^ cells/kg for subcutaneous use). In our previous study, the therapeutic dosage of hUC-MSCs for transplantation including acute lung injury in rats, was 0.4 ×  10^6^ cells/kg (Cui et al., 2022). Based on these data, 3.0 × 10^6^ cells/kg and 3.0 × 10^7^ cells/kg were selected as the low and high intravenous doses, respectively. A dose of 3.0 × 10^7^ cells/kg was selected as the subcutaneous dose. hUC-MSCs were administered twice a week for 3 weeks at D1, D3, D8, D10, D15, and D17. The volume of doses was 2.5 mL/kg for the first four doses. For the latest two doses, the volume of doses was 5.0 mL/kg for the control, IVL, and IVH groups and 2.5 mL/kg for SC group (Figure 1).

### 2.4 Clinical examination

Observations regarding the general health, clinical abnormal symptoms and mortality were recorded daily. Body weight, body temperature and food consumption were monitored weekly. Food consumption was graded by percentage of feed as A∼F: where *A* = 100%, *B* = 75%∼100%, *C* = 50%∼75%, *D* = 25%∼50%, *E*=<25%, *F* = 0%. The hematology, serum biochemistry, serum immunoglobulin (IgG and IgM), complement (C3 and C4), peripheral blood lymphocyte (PBLC) subtype (CD3^+^, CD4^+^, and CD8^+^), and electrocardiogram were monitored the day after the 2nd dosing (D4), at the end of the dosing phase(D30) and at the end of the recovery period (D59). Ophthalmology was performed at D30 and D59. Urine examination and histopathological evaluation were performed at the end of the drug phase (D31) and recovery period (D60). All major organs including the heart, liver, spleen, lung, kidney, brain, thymus, and mesenteric lymph nodes, were removed and fixed in 4% paraformaldehyde for microscopic observation. Lesions were graded as follows: -, none; +, minimal; ++, mild; +++, moderate, ++++, marked, or +++++, severe).

### 2.5 Toxicokinetic study

The toxicokinetic profile of hUC-MSCs was determined by real-time quantitative PCR detection of the SRY gene in blood and tissues, of which the DNA sequence is specific to *Homo sapiens*. Genomic DNA was extracted for qPCR analysis of the SRY gene as described previously (Wang et al., 2018). The number of SRY gene copies was determined to accurately quantify the number of hUC-MSCs. The toxicokinetics (TK) of hUC-MSCs in cynomolgus monkeys were determined at the first or last administration. Blood samples (0.5∼0.8 mL) were taken prior to hUC-MSC administrations and 5 min, 1, 4, 8, 24, and 48 h after administration.

### 2.6 Statistical analysis

In the present study, we used the mean ± standard deviation (SD) to describe the quantitative data such as the weight and growth rate, body temperature, hematology, biochemistry, electrocardiogram, immunology, and organ weights and ratios. Statistical significance was analyzed by one-way ANOVA using SPSS 17.0 software. Grade data such as the food intake and urine were compared using Kruskal-Wallis nonparametric tests. Differences of *p* ≤ 0.05 were considered significant.

## 3 Results

### 3.1 Isolation and characterization of hUC-MSCs

**FIGURE 2:**
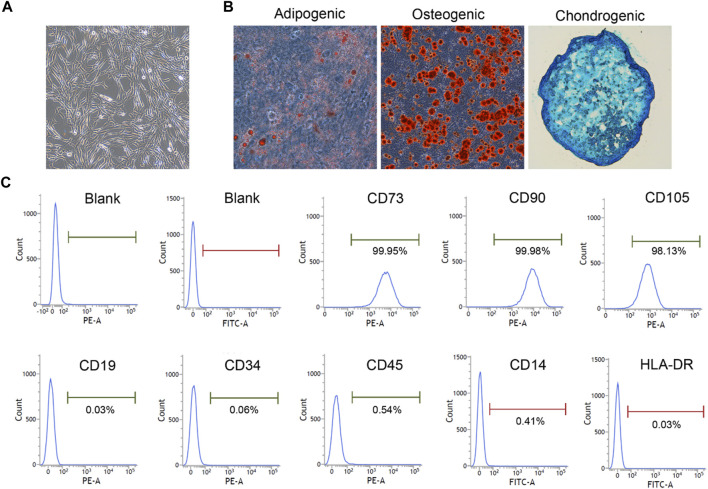
hUC-MSC was characterized by morphology, differentiation capacity and surface markers (×100). **(A)** The morphology of HUCMSC. **(B)** Differentiation ability test. Osteogenic, adipogenic, and chondrogenic differentiation capacity were detected using Alizarin Red, Oil Red O, and Alcian Blue staining, respectively (×100). **(C)** Phenotypic analysis. Surface markers were detected by flow cytometry.

hUC-MSCs were isolated from umbilical cord tissues and cultured until passage 5. Cells were characterized based on their morphology, mesenchymal lineage differentiation, and surface markers. The UC-MSCs exhibited a typical spindle-shaped morphology (Figure 2A). After *in vitro* induction, the UC-MSCs were successfully differentiated into mesenchymal derivatives, including osteoblasts, adipocytes, and chondrocytes (Figure 2B). Furthermore, as determined using flow cytometry, cells were positively stained for CD73, CD90, and CD105, and did not express hematopoietic markers, CD19, CD34, CD45, CD14 and HLA-DR (Figure 2C). These results suggest that hUC-MSCs fulfill the criteria for typical MSC as outlined by the International Society for Cellular Therapy guidelines (Dominici, Le Blanc, Mueller et al., 2006).

### 3.2 General observation

For the clinical observation, no adverse effects were observed in control, IVL and SC groups. During the first and second administration, IVH group animals showed no abnormal reaction. About 2 or 3 min after the 3rd, 4th dosing, parts of monkeys exhibited coma (1/10, 3/10), decreased breath rate, dilated pupil, low mobility (shown in Supplementary Table S8). Considering the seriousness of the adverse effects and even the possibility of death, we diluted the cell concentration and slowed down the infusion speed during the last two administrations. After the 5th, 6th dosing, none of the monkeys were comatose, and only 5 or 7 monkeys showed a milder degree of hypokinesia, hypopnea, dilated pupils, closed eyelids, or hypoplasia of fatigue within 10 min after administration. Significant improvement in adverse effects after adjusting the dosing schedule. Subcutaneous induration at the injection site (dorsal skin) was observed in all of animals in SC group and disappeared within 1 week after the last administration.

### 3.3 Body weight, food consumption, and body temperature changes

**FIGURE 3:**
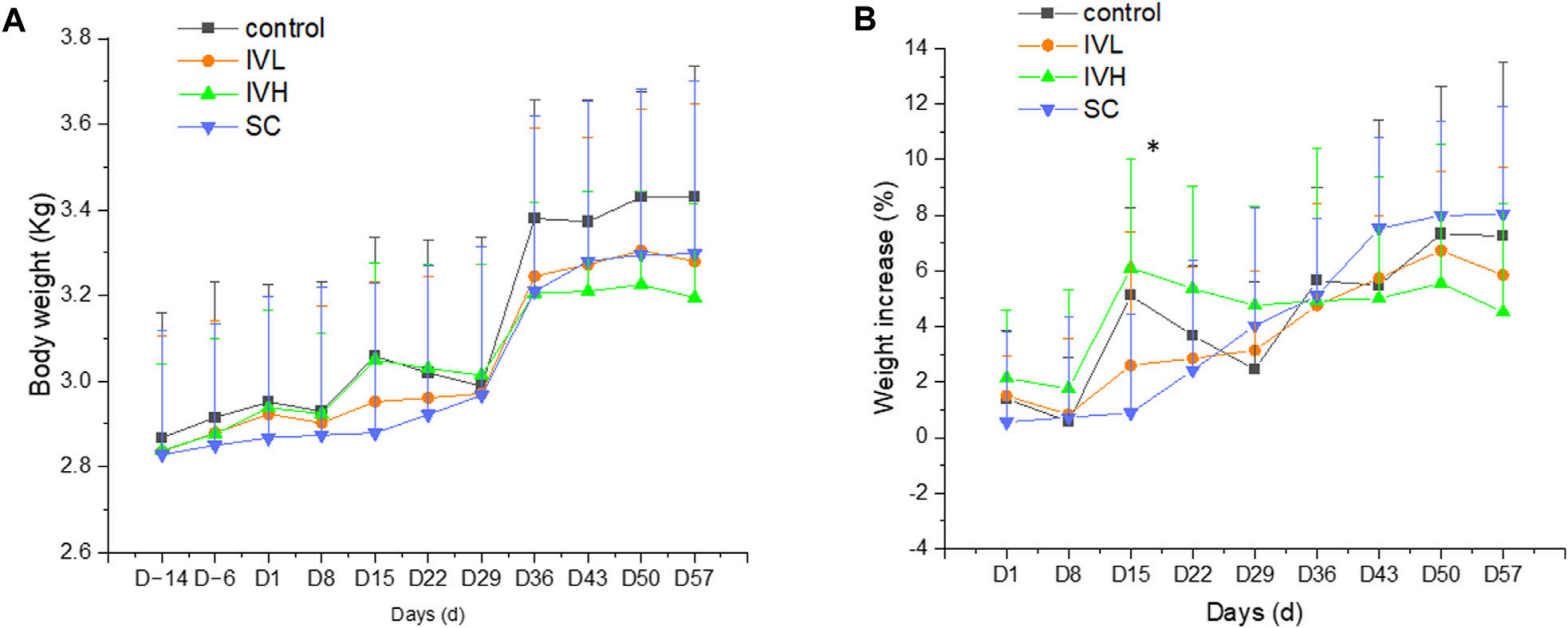
Changes in body weight and the rate of body weight **(A,B)** increase in cynomolgus monkeys after 6 repeated hUC-MSC administrations. *n* = 10 at D-14∼D29, *n* = 4 at D36∼D57 in each group vs. the control group (*p* > 0.05).

On D15, the rate of body weight increase in the SC group was moderately lower than that in the control group. On D3 and D17, food consumption in the IVH group was lower than that in the control group (*p <* 0.05, *p <* 0.01). On D8, body temperature in the SC group was lower than that in the control monkeys (*p* < 0.01). These minor fluctuations are considered to be of no toxicological significance (Figure 3; Supplementary Tables S1, S2).

### 3.4 Haematology and biochemistry findings

**FIGURE 4:**
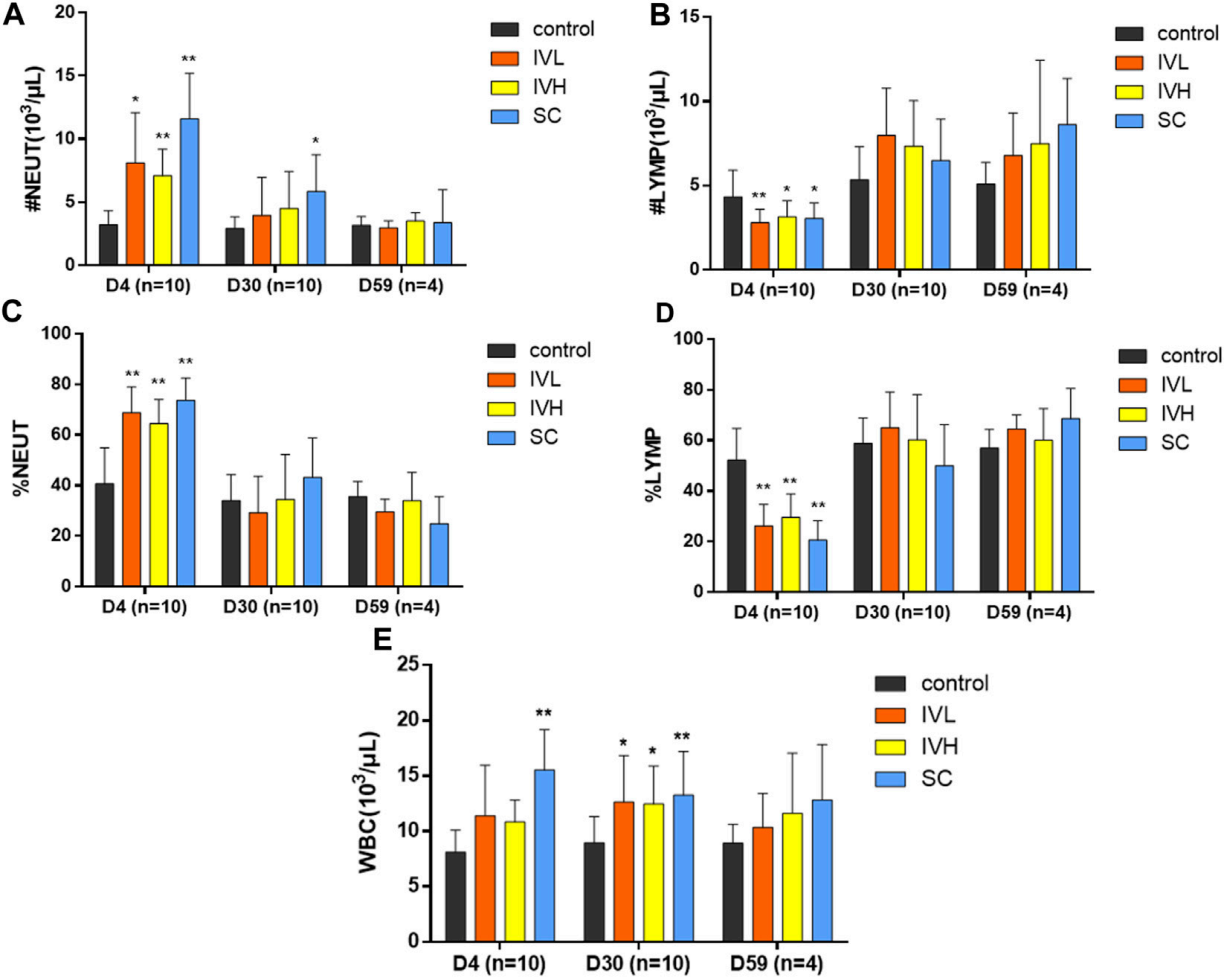
The effects of hUC-MSC administration on #NEUT, %NEUT, #LYMPH, %LYMPH, and WBC counts **(A–E)** in cynomolgus monkeys. *p < 0.05, **p < 0.01, vs. the control group.

On D4 (the second dosing examination), the neutrophil count (#NEUT), percentage of neutrophils (%NEUT), and white blood cells (WBCs) in the IVL, IVH, and SC groups were higher than those in the control group, whereas the lymphocyte count (#LYMPH), percentage of lymphocytes (%LYMPH), red blood cells (RBCs), haemoglobin (HGB), and hematocrit (HCT) were lower than those in the control group (*p* < 0.05, *p* < 0.01). At the end of the dosing phase, #NEUT, WBCs, the percentage of basophiles (%BASO), eosinophils (#EOS), #BASO, reticulocytes (#RETIC), and %RETIC in the IVL, IVH, and SC group were higher than those in the control group, while the percentage of monocytes (%MONO) was lower than that in the control group (*p* < 0.05, *p* < 0.01) (Figure 4; Supplementary Table S3).

On D4 (the second dosing examination), total protein (TP), albumin (ALB), and Ca^2+^ in the SC group were lower than those in the control group, and Na^+^ was higher than that in the control group (*p* < 0.05, *p* < 0.01). At the end of the dosing phase, GLU, ALB, A/G, Cl^−^, and Ca^2+^ in SC group was lower than those in the control group, and TG was higher than that in the control group (*p* < 0.05, *p* < 0.01). However, the magnitude of these changes was relatively small, and they are considered to be of no toxicological significance. At the end of the recovery period, no significant difference in hematology and biochemistry changes was found in any group compared to the control group (Table 1).

**TABLE 1:** Blood biochemistry analysis in cynomolgus macaques treated with hUC-MSCs in repeat-dose toxicity study.

Parameter	After 2nd administration (*n* = 10)				End of the dosing phase (*n* = 10)				End of the recovery period (*n* = 4)			
	Control	IVL	IVH	SC	Control	IVL	IVH	SC	Control	IVL	IVH	SC
ALT (IU/L)	62.07 ± 16.62	125.72 ± 74.07	98.55 ± 42.14	99.45 ± 60.82	45.11 ± 13.1	37.62 ± 7.78	45.29 ± 12.05	43.97 ± 14.24	48.07 ± 8.06	33.61 ± 13.41	38.42 ± 8.9	46.33 ± 12.86
AST (IU/L)	63.03 ± 11.6	159.41 ± 108.28	92.34 ± 69.41	95.54 ± 72.47	58.54 ± 25.87	56.74 ± 12.45	56.48 ± 26.05	50.13 ± 12.65	53.78 ± 15.18	46.24 ± 11.19	43.28 ± 12.43	47.04 ± 11.69
T.BIL (μmol/L)	4.667 ± 1.233	4.439 ± 1.057	5.117 ± 2.156	3.84 ± 1.09	3.774 ± 0.948	3.1 ± 0.758	4.545 ± 2.632	3.068 ± 0.741	4.734 ± 1.526	3.302 ± 0.399	4.969 ± 4.154	3.564 ± 0.483
ALP (IU/L)	524.99 ± 107.18	443.57 ± 96.53	514.93 ± 84.64	492.97 ± 144.56	556.58 ± 194.59	436.78 ± 166.13	545.22 ± 176.88	448.15 ± 127.06	751.9 ± 113.73	523.27 ± 227.47	640.35 ± 250.43	571.51 ± 109.94
r-GT (IU/L)	70.83 ± 23.66	59.02 ± 25.13	69.53 ± 22.67	58.15 ± 11.56	82.42 ± 28.2	70.33 ± 36.88	78.92 ± 31.93	70.42 ± 17.86	100.81 ± 37.26	86.26 ± 62.85	74.08 ± 20.8	76.79 ± 12.64
CK (IU/L)	392.9 ± 234.8	1420 ± 1380.7	2084.5 ± 3215	954.6 ± 1407.3	259.1 ± 127.8	255.5 ± 72.2	481.1 ± 585.6	252.1 ± 91.1	275.7 ± 91.8	202.1 ± 68.7	271.4 ± 102.3	302.5 ± 92.4
BUN (mmol/L)	6.99 ± 1.289	7.229 ± 1.187	7.058 ± 1.476	7.809 ± 1.184	7.272 ± 1.717	6.592 ± 0.989	6.555 ± 1.412	7.372 ± 1.229	7.565 ± 2.194	6.502 ± 1.36	6.647 ± 1.137	7.482 ± 1.088
Crea (μmol/L)	82.24 ± 9.11	84.34 ± 11.03	84.93 ± 5.4	84.57 ± 14.52	89.48 ± 11.41	85.93 ± 13.5	83.77 ± 7.63	87.11 ± 15.71	95.4 ± 10.71	94.25 ± 13.92	95.15 ± 16.61	101.95 ± 10.9
T.P (g/L)	85.44 ± 4.37	82.58 ± 4.68	82.89 ± 4.07	78.23 ± 5.31**	80.74 ± 6.05	82.67 ± 4.74	80.34 ± 5.3	79.42 ± 5.85	80.9 ± 6.37	79.66 ± 4.44	83.09 ± 8.09	77.52 ± 3.97
ALB (g/L)	43.36 ± 2.22	41.86 ± 1.69	42.02 ± 1.67	39.92 ± 2.15**	41.92 ± 1.93	41.57 ± 1.6	41.25 ± 1.46	39.13 ± 2.59*	42.24 ± 1.82	41.4 ± 2.28	42.26 ± 2.89	41.75 ± 2.14
GLO (g/L)	42.09 ± 3.78	40.72 ± 3.79	40.87 ± 4.17	38.31 ± 4.36	38.82 ± 5.36	41.1 ± 3.76	39.09 ± 4.6	40.28 ± 3.97	38.66 ± 4.9	38.26 ± 2.26	40.83 ± 6.18	35.76 ± 2.83
A/G	1.04 ± 0.1	1.03 ± 0.09	1.04 ± 0.13	1.05 ± 0.11	1.1 ± 0.14	1.02 ± 0.08	1.07 ± 0.12	0.98 ± 0.08*	1.1 ± 0.11	1.08 ± 0.03	1.05 ± 0.16	1.17 ± 0.09
GLU (mmol/L)	5.15 ± 1.457	5.043 ± 0.799	4.698 ± 1.434	4.855 ± 1.348	5.001 ± 2.203	4.046 ± 1.002	3.888 ± 0.986	3.422 ± 0.746*	3.768 ± 1.202	4.35 ± 1.76	3.486 ± 0.161	4.818 ± 2.371
T.CHO (mmol/L)	3.589 ± 0.735	3.392 ± 0.713	3.458 ± 0.397	3.448 ± 0.675	3.652 ± 0.872	3.406 ± 0.909	3.238 ± 0.449	3.675 ± 0.906	3.526 ± 0.491	3.26 ± 0.342	3.762 ± 0.106	3.258 ± 0.38
TG (mmol/L)	0.599 ± 0.084	0.605 ± 0.121	0.592 ± 0.093	0.66 ± 0.135	0.336 ± 0.09	0.366 ± 0.156	0.347 ± 0.075	0.476 ± 0.149*	0.464 ± 0.077	0.366 ± 0.095	0.451 ± 0.098	0.471 ± 0.089
K^+^(mmol/L)	4.55 ± 0.26	4.89 ± 0.55	4.83 ± 0.3	4.4 ± 0.4	4.48 ± 0.43	4.59 ± 0.43	4.81 ± 0.34	4.35 ± 0.35	4.6 ± 0.77	4.55 ± 0.42	4.57 ± 0.62	3.89 ± 0.44
Na^+^(mmol/L)	149.1 ± 1.5	150.8 ± 3.1	150.9 ± 2.4	150.7 ± 1.2*	148 ± 1.2	147.6 ± 2.2	148.4 ± 0.9	148.7 ± 1.1	150.5 ± 0.8	151.7 ± 3.4	153.6 ± 5.4	152.8 ± 1.5*
Cl^−^(mmol/L)	107 ± 1.4	107.1 ± 2.8	107.8 ± 2.4	108.3 ± 1.8	106.3 ± 2.5	101.9 ± 4	102.1 ± 5.7	101.7 ± 5.5*	109.6 ± 2.2	110 ± 0.9	110.9 ± 2.7	111.3 ± 2.1
Ca(mmol/L)	2.25 ± 0.09	2.22 ± 0.1	2.24 ± 0.09	2.16 ± 0.07*	2.48 ± 0.09	2.48 ± 0.05	2.47 ± 0.1	2.37 ± 0.12*	2.46 ± 0.13	2.42 ± 0.04	2.51 ± 0.18	2.42 ± 0.13

Note: Data are expressed as the means ± SD. *, ** indicate a statistically significant difference at *p* < 0.05 and *p* < 0.01 when compared to the control group; Abbreviations: ALT, alanine aminotransferase; AST, aspartate aminotransferase; TP, total protein; ALB, albumin; TBIL, total bilirubin; ALP, alkaline phosphatase; r-GT, r-glutamyltransferase; GLU, glucose; BUN, blood urea nitrogen; Crea, creatinine; CHO, cholesterol; TG, triglyceride; CK, creatine phosphokinase; GLO, globulin; A/G, albumin/globulin ratio.

On D4 (the second dosing examination), no significant difference in ECG was found in any hUC-MSCs-treated group compared to the control group. At the end of the dosing phase, the PR interval (PR) and P wave duration (Pdur) in the SC group were lower than those in the control group (*p* < 0.05). At the end of the recovery period, the ST-segment level in IVL group was lower than that in the control group (*p* < 0.05) (Supplementary Table S4). However, these changes fluctuated and fell within the laboratory historical range and were believed to be irrelevant to the hUC-MSCs treatment. There were no significant differences in ophthalmic and urine examinations in hUC-MSC-treated groups compared with the control group throughout the study (data not shown).

### 3.5 Immunological indicators

**FIGURE 5:**
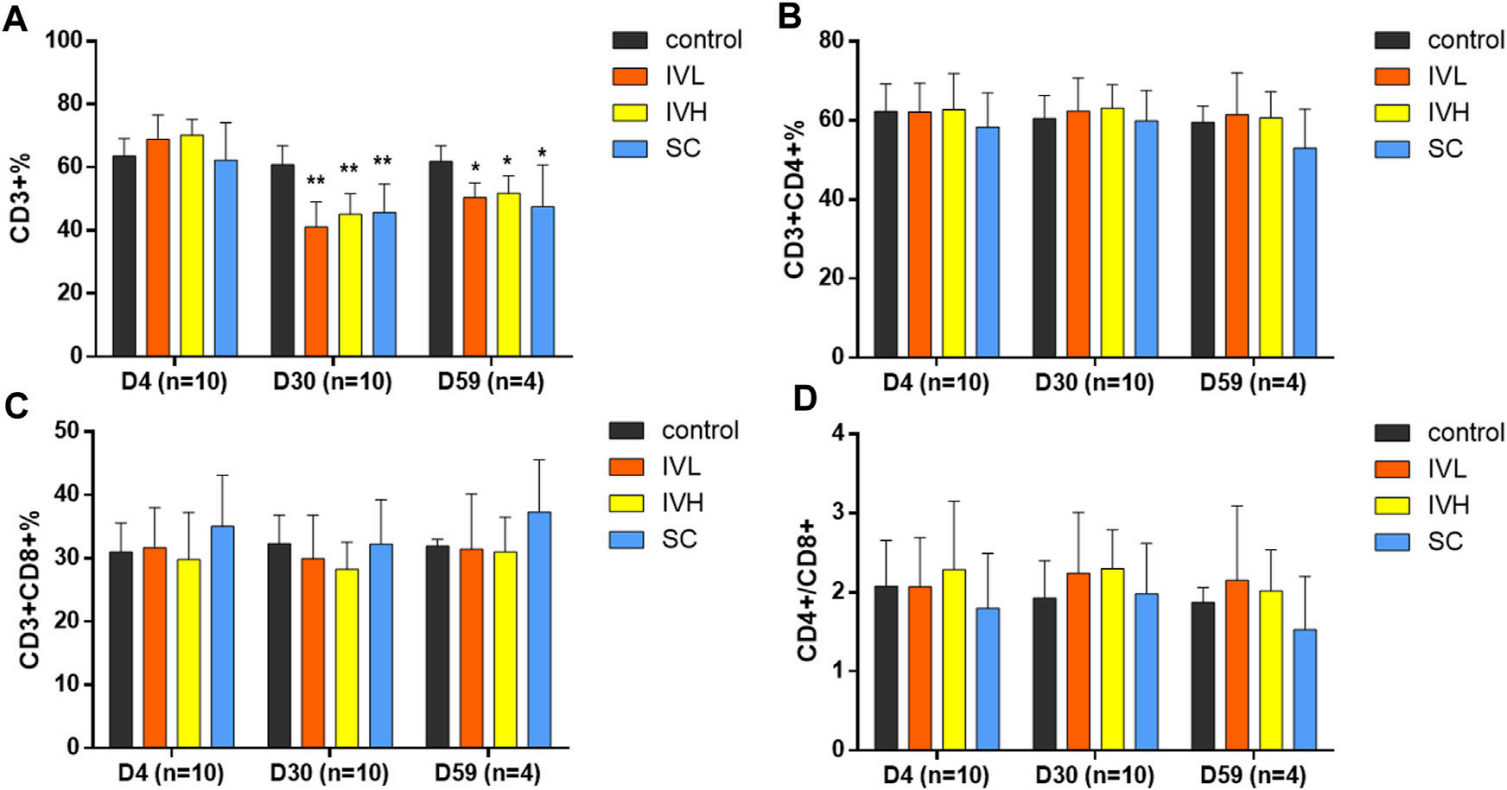
The effects of hUC-MSC administration on the CD3+%, CD3^+^CD4+%, CD3^+^CD8+%, and CD4+/CD8+, **(A–D)** in cynomolgus monkeys. *p < 0.05, **p < 0.01, vs. the control group. D4 (*n* = 10). D30 (*n* = 10), D59 (*n* = 4).

Analysis of T-cell subsets showed that the subpopulations of CD3^+^ T lymphocytes in the IVL, IVH and SC groups were significantly decreased when compared to those in the control group at the end of the dosing phase (*p* < 0.05, *p* < 0.01). The descent rates of CD3^+^ cells were 32.5%, 25.8% and 24.9%. At the end of the recovery phase, the number of CD3^+^ T lymphocytes in the IVL and IVH groups were significantly lower than that in the control group (*p* < 0.05). The descent rate of CD3^+^ was 18.4%, and 16.3%. The percentages of CD3^+^CD4^+^ subsets and CD3^+^CD8^+^ subsets and the CD4+/CD8a+ratio showed no changes. No significant changes in IgG, IgM, C3, or C4 levels were found at the second dosing inspection, at the end of the dosing phase or in the recovery period (Figure 5; Supplementary Table S5).

### 3.6 Histopathological findings

No significant macroscopic changes were found in major organs between the control and hUC-MSC treated group. There were no significant changes in organ weights or organ coefficients, except that the spleen and spleen coefficient were significantly increased in IVH group cynomolgus macaques (*p* < 0.05) (Supplementary Table S6).

**FIGURE 6:**
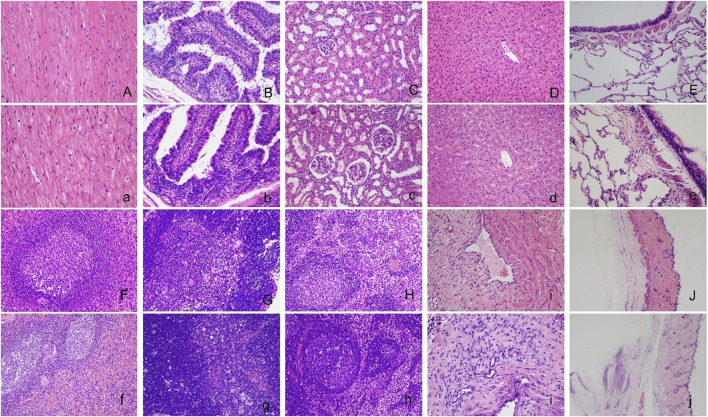
Histopathological images of the major organs in cynomolgus macaques after repeated administration of hUC-MSCs. No significant abnormal changes were found in the brain **(A, a)**, jejunum **(B, b)**, kidney **(C, c)**, liver **(D, d)**, lung **(E, e)**, spleen **(F, f)**, thymus **(G, g)**, lymph node **(H, h)** or vein infusion site **(I, i)** between the control **(A∼I)** and IVH groups **(a∼i)**. Images of the subcutaneous injection site of the control **(J)** and SC group **(j)**. Serious damage at the subcutaneous injection site in the SC group compared with the control group. H&E staining (×200) for A to i and (×20) for **(J)** and **(j)**.

No significant abnormal changes were found in the brain, jejunum, kidney, liver, lung, spleen, thymus, or lymph nodes after repeated administration of hUC-MSCs. Histological changes at the vein infusion site including mild granulation tissue proliferation were observed at the end of the dosing phase. The similar characteristics and severity between the control, IVL, and IVH groups, suggest that the changes were not related to hUC-MSCs administration, may be caused by mechanical venipuncture. Consistent with the macroscopic subcutaneous induration of the back skin, focal granulomatous inflammation lesions were observed at the subcutaneous injection site in SC group animals (Figure 6). After 4 weeks of recovery, the histological damage described above was alleviated.

Most of the cynomolgus monkeys administered hUC-MSCs did not show any pathological lesions. Spontaneous background pathological findings including focal cellular infiltration in the heart, kidney, and bladder and microgranuloma in the liver were identically observed in each group (Supplementary Table S7).

### 3.7 Toxicokinetic study

At 1 h after the first infusion, the human SRY sequence was detected from 1/8 monkeys (150 copies/μL) in IVL monkey blood. At 5 min and 1 h after the first infusion, the human SRY sequence in 7/8 IVH monkey blood was detected (56∼342 copies/μL). The human SRY sequence was not detected in the blood from all 8 SC group monkeys.

For the last infusion, the human SRY sequence was below the limit of quantification in all 8 IVL monkey blood samples. At 5 min and 1 h after the last infusion, the human SRY sequence in 6/8 IVH monkey blood samples were detected (50∼206 copies/μL). At 1 h after the infusion, the human SRY sequence was detected (66∼149 copies/μL) in 3/8 SC monkey blood samples.

We found that the infused hUC-MSCs disappeared in blood within 1 h, and the rapid decrease in SRY gene copies is consistent with the previous literature(Wang et al., 2012). In total, the concentration in the IVH group was higher than that in the IVL group and the SC group (Table 2).

**TABLE 2:** hUC-MSC SRY gene copy number in cynomolgus monkey blood (copies/μL).

Date	Group	Time (h)				Animal				
			1	2	3	4	5	6	7	8
D1	IVL	0.083	0	0	0	0	0	0	0	0
		1	0	0	0	150	0	0	0	0
		4/8/24/48	0	0	0	0	0	0	0	0
	IVH	0.083	56	342	0	0	87	0	0	0
		1	0	0	124	0	171	121	204	102
		4/8/24/48	0	0	0	0	0	0	0	0
	SC	0.083/1/4/8/24/48	0	0	0	0	0	0	0	0
D17	IVL	0.083/1/4/8/24/48	0	0	0	0	0	0	0	0
	IVH	0.083	0	176	50	Miss	51	Miss	0	0
		1	0	0	0	0	59	62	145	206
		4/8/24/48	0	0	0	0	0	0	0	0
	SC	0.083	0	0	0	0	0	0	0	0
		1	66	123	0	149	0	0	0	0
		4/8/24/48	0	0	0	0	0	0	0	0

Note: 4/8/24/48 represent the sampling time point as 4, 8, 24, and 48 h, 0.083/1/4/8/24/48 represent the sampling time point as 0.083, 1, 4,8,24, and 48 h.

## 4 Discussion

Our results demonstrated the safety profile of repeat-dose hUC-MSCs in cynomolgus monkeys by the intravenous and subcutaneous routes. The six administrations of hUC-MSCs produced no systemic toxicity changes including weight, temperature, hematology, clinical chemistry, ECG, and pathology.

It was previously reported that the intravascular infusion of MSCs caused pulmonary embolism or vascular embolism in animal experiments and clinical trials (Lee, Seo, Pulin et al., 2009; Jung, Kwon, Choi et al., 2013; Liu, Chiang, Hung et al., 2017; Li, Huang, Zhang et al., 2022). When MSCs were infused into the systemic circulation rapidly, most of the MSCs were immediately entrapped in the lung. MSCs formed emboli that blocked small pulmonary vessels. In our present study, The increasing cynomolgus monkeys in the IVH group rapidly developed coma symptoms 2–3 min after the 3rd doses. To improve animal welfare and avoid possible death, we doubled the hUC-MSC infusion volume from 2.5 mL/kg to 5.0 mL/kg in the last 2 administrations, the infusion speed was decreased from 42.8 ± 4.5 (×10^6^ cells)/min to 18.3 ± 2.4 (×10^6^ cells)/min. Frailty symptoms such as low respiratory rate, low mobility, mydriasis, exhaustion, and eyelid closure were significantly alleviated. Importantly, none of the IVH group animals lost consciousness. Our study showed that slow infusion eliminated coma symptoms. To explore the cause of the weakness symptoms induced by hUC-MSCs infusion, an extra ECG examination was conducted on all 10 monkeys in the IVH group before and 10 min after the 5th hUC-MSCs dose. In contrast to the normal ECG at pre-dosing, hUC-MSCs caused a rapid ST segment depression and T wave amplitude increase (Supplementary Figure S1), which is consistent with pulmonary embolism (Konstantinides, Meyer, Becattini et al., 2019). We speculated that the transient coma in the cynomolgus monkeys may be due to the acute pulmonary embolism caused by rapid infusion of MSCs. However, the definitive diagnosis of acute pulmonary embolism requires a particular examination, such as a chest CT scan, computed tomographic pulmonary angiography (CTPA) and blood gas analysis. No lung pathological damage was found in cynomolgus monkeys. All these results indicated that the coma symptoms were infusion velocity dependent, not a toxicological effect of hUC-MSCs. According to the literature, MSC infusion at a speed of 2 × 10^6^ cells/min in patients and 3∼4 × 10^6^ cells/min in cynomolgus monkeys did not cause abnormal symptoms(Wang et al., 2012; Weiss, Casaburi, Flannery et al., 2013; Le Thi Bich et al., 2020). In future animal experiments and clinical trials, the slow-speed infusion of hUC-MSCs is recommended.

In the present repeated-dose study, psychiatric symptoms such as coma and decreased respiratory rate were observed in monkeys given high doses and high concentrations of hUC-MSCs intravenously. A large amount of stem cells entered the systemic circulation and blocked the pulmonary vasculature, leading to a decrease in heart rate and blood pressure. Then inadequate blood supply to the brain leads to rapid loss of consciousness and dilated pupils, which may be a sign of autonomic dysfunction. TEMCELL, a drug approved and marketed for the treatment of MSCs in GVHD, is listed here to help understand the adverse effects of MSCs. Intravenous administration of TEMCELL resulted in pulmonary embolism in a preclinical study of 4-week repeated dosing in mice and 13-week repeated dosing in rats(PMDA Japan, 2015). Decreased activity, loss of righting reflex, red urine, pale skin over the body, hunched posture, and salivation was observed both in mice and rats. The pathological examination revealed the presence of cellular embolism and thrombus in the lung which suggested the pulmonary embolism existed. TEMCELL distribution study in SCID mice revealed higher concentrations of TEMCELL in the lungs than in other organs within 2 h of perfusion. The concentration at 24 h post-infusion decreased sharply to approximately 1/100th of that at 2 h. The concentrations of TEMCELL in the spleen and liver increased from 2 h, suggesting that stem cells were redistributed from the lung to spleen, liver or other peripheral organs via the bloodstream. High concentrations of MCSs predispose the lungs to the formation of occlusions. The rapid redistribution of stem cells from the lungs may account for the rapid recovery from loss of consciousness. It has been reported in the literature that administration of potent vasodilators, such as sodium nitroprusside or nitric oxide, increases the outflow of MSCs from the lungs and alleviates pulmonary embolism (Boltze et al., 2015). Our present study demonstrated that lowering cell concentrations and slowly infusing MSCs is an effective method to reduce the severity of pulmonary embolism in monkeys. This is consistent with the alleviation of pulmonary embolism achieved by vascular expansion in the above reference.

The symptoms of coma, decreased respiration, dilated pupils, decreased activity, and mental exhaustion in monkeys after repeated doses of hUC-MSCs (on the 3rd/4th dose prior to dilution of the concentration) may be typical of those associated with pulmonary embolism, consistent with many reports in the literature (Jung et al., 2013; Tatsumi, Ohashi, Matsubara et al., 2013; Li et al., 2022). However, it is also necessary to exclude whether this is due to an allergic reaction. On the one hand, we found no significant changes in indicators of immunotoxicity, including IgG, IgM, complement C3, C4, CD4^+^, and CD8^+^ T-cell subsets, in the present study. On the other hand, we also performed an active systemic allergy study (ASA) after intravascular injection of hUC-MSCs in guinea pigs. At 14 and 21 days after the third sensitization administration, each animal was injected intravenously with 1 × 10^6^ cells of hUC-MSCs in a provocation test, which did not show any signs of allergic reaction (Supplementary Table S9). In addition, based on the results of our non-clinical studies, the hUC-MSCs candidate has received clinical trial approval from National Medicinal Products Agency (NMPA, China, registration number: CXSL2300221). In summary, the possibility of hUC-MSCs inducing allergic reactions is very low. The existing adverse reactions in the high-dose group may be due to pulmonary embolism introduced by intravenous injection of high concentrations of stem cells. Therefore, extra attention should be paid to the infusion rate, cell concentration, and cell homogeneity in the next step of intravenous human clinical trials to prevent infusion reactions.

The immunosuppressive properties of MSCs have been quite well documented over the last few years. The immunosuppressive effects of hUC-MSCs underlined the potential of MSC transplantation therapy against immune disease. As found in a sepsis model in rats and dermatitis model in canines, MSCs administration increased Treg levels and function, inhibited T-cell proliferation, and exerted immunosuppressive roles (Chao et al., 2014; Byeon, Lee, Kim et al., 2016; Le Thi Bich et al., 2020). However, there are few reports on hUC-MSC immunoregulatory activity in healthy animals. Our study showed that hUC-MSCs infusion significantly increased NEUT, decreased LYMPH and T cells (CD3^+^ cells), and significantly increased the spleen weight and weight coefficient in cynomolgus monkeys, showing its immunoregulatory effect, which was consistent with a previous study (He, Ruan, Yao et al., 2017; Le X et al., 2020). For the reason of potent immunosuppressive roles, attention should be given to increased risk of diseases such as bacterial and viral infection and new-onset or relapsed tumors in hUC-MSC clinical applications. A literature confirmed that MSCs inhibits the activation of T cells in the cell G0/G1 phase without causing T cell apoptosis (Di Nicola, Carlo-Stella, Magni et al., 2002). Our histological examination of the thymus, spleen and lymph nodes shown no hUC-MSC-related toxicological changes. In summary, we reasoned that hUC-MSC be sufficient to impact negatively upon lymphocyte without inducing pathological alteration.

No obvious systemic toxic reactions were found in the monkeys in the subcutaneous injection group, and only recoverable subcutaneous hard nodules and focal granulomatous inflammatory foci at the injection site were observed, which were considered to be short-term localized inflammatory lesions formed between the stem cells after subcutaneous injection and subcutaneous tissue cells. Based on this result, it is predicted that there may be no major safety risks associated with subcutaneous administration during subsequent clinical studies, except for possible localized inflammatory symptoms at the injection site.

Non-clinical safety evaluations are critical in the drug development process for human risk assessment and to support clinical development. Based on the results of the present monkey study and other nonclinical studies in other animal species such as severely immunodeficient mice, rabbits, and guinea pigs, the hUC-MSCs candidate has received clinical trial approval from National Medicinal Products Agency of China. Based on the results of the present study, the no observed adverse effect level (NOAEL) was 3 × 10^6^ cells/kg hUC-MSCs for intravenous infusion in cynomolgus monkeys and 3 × 10^7^ cells/kg hUC-MSCs for subcutaneous injection, which were equivalent to 1.5 and 30 times the clinical dose. In summary, the present study declared the preliminary safety of hUC-MSCs, but close monitoring of hUC-MSCs for adverse effects, such as coma induced by intravenous infusion, is warranted in future clinical trials.

## Data Availability

The original contributions presented in the study are included in the article/Supplementary Material, further inquiries can be directed to the corresponding authors.
